# A Historical Poem

**Published:** 1883-12

**Authors:** J. F. Siddall

**Affiliations:** Oberlin


### A Historical Poem.

BY J. F. SIDDALL, OBERLIN.

Read at the Ohio State Dental Society, Nov. 1st, 1883.

### PRELUDE.

Early in life I became somewhat dissipated. Among other bad habits which were a great affliction to my friends, I contracted the poetry habit. Inasmuch as I had been prevailed upon to sign a pledge to forever abstain from the use of tobacco and grog, it was thought best not to attempt too much with the boy, and the thing was let run for self-cure, which was supposed to have been accomplished. After many years, under great provocation, there came a relapse. The circumstances were these: A patron, who for years had been taking the liberty of getting his dentistry done at my office gratis, had a set of *permanent teeth* made. When ready, I wished to inform him of two things; first, that the teeth were made; and secondly, that they must be paid for before he could have them. I seized my pen, and the old spirit came upon me, very much to the disgust I fear of my patient. Here it is:

Your rubber and crockery are ready for delivery,
Come on with the tin and have them put in.

Those teeth lay in my drawer a year and five months, in which time the temporary set *failed* and came to the rescue.

PREFACE.

I believe it is the fashion for a book to have a preface, and for a great poet to apologize; so, of course, I must be in fashion. The origin of what you are about to hear is the result of an accident. And I can assure you that Dr. Taft will be careful never to *do so again*, when he sees what has come of his mistake.

Understanding that this meeting was to be the first week in October as advertised in the REGISTER, and no programme coming to hand as usual; and the one previously sent at the beginning of the year, being *lost, of course*; I said to myself I must be prepared to take my part in this meeting, and in the absence of a list of subjects, I wound up my old machine and let her drive. So here she goes:

THE OHIO STATE DENTAL SOCIETY.

Our State Society's been getting unsteady,
I suppose it's because she's seventeen already,
And will soon be of age and is letting us know it
In time, for to see her just up and go it.

The last end of the teens is an age so uncertain,
That you never are sure what's behind the curtain,
You may think all is right, but dare you not to tell it,
For its little you know as to what may develop.

For many long years we have met in December,
As most who are here very well can remember;
When society reached 17 she knock'd all that over,
And said to us "boys better come in October;"
And now, we are here at the time she has told us
In our Sunday's best—come my darling, behold us.

In June '66 we had our beginning,
As good as the best was our underpinning,
We started off well with a constitution
And a code of ethics at our very first meeting.

Our code was the first ever known on record,
And it stands to-day with a fame unchecker'd,
Being used as a model with much propriety
At the next great gathering of the National society,
At which, with a sleight and trifling variation,
It was made to run the whole dental nation.
Then all the States got their codes and fixed 'em,
Not knowing that Ohio was the goad that had prick'd 'em.

As for law we dealt in that as well as in gospel,
Ohio is known as the foremost apostle
Of legal enactment to regulate dentists;
Right early our society to this work has sent us.

If Ohio's been ahead in all such doings
About codes, and laws, and all such brewings,
You may be sure that somebody's to blame for it,
And a one man power is about the name for it.
I might go farther back than the record,
And name the guilty man suspected;
But no, 'twont do, he has been forgiven,
And his name should rest in sweet oblivion.

In '66 we began the foundation,
Which, in '68 had reached its completion,
And our law then stood as a wall around us
When it gathered us in wherever it found us.

In '73 was a rousen big harvest,
The crop came in from regions the farthest,
And brought the best price ever known among us
Just twenty-five dollars, a head in Columbus.

We made the board sweat that year I can tell you,
But they sweat us when they said "we won't sell you,"
"You're not worth the price the State puts on you,
Just keep your ill-gotten gain and begone you."

From one to two hundred old chaps and young ones,
Most all with heads up but a few who hung them,
Were seen going home each man in position
With a flee in his ear as to State prohibition.

Sure enough the time soon came when the women
Said "give us more law to stop the men's sinnen;"
And more than three hundred thousand voters
Marched up to the polls and obeyed their orders.
And now let me make right here a prediction
As sure as our law to stop quacks is no fiction,
And works worth a cent to prohibit tooth pullers
So sure you can stop Ohio's rum-sellers.
But *O!* how hard it is to be willing
To ever give up that you can't for a shilling,
Get a set of store teeth or a good whisky toddy
In spite of law without fear of anybody.

I'm ahead of my story and came near forgetting
On one cold night when society was setting,
There arose a new financial reformer,
To put prices down by the name of Warner.

This Warner they say, just raised such a rumpus,
It shook the whole State from center to compass
Till soon you could get the Board to pass you;
For a fee, if once you had offered, they'd sass you.

But Warner's head, to be sure, was level,
For well he knew that a skulking devil
Had been prowling around and bleeding freely,
For the proof of which was the throat of Keely.

Now, these were days of war and fighting,
And one big shark did all the biting
Required to keep a dentist scratching,
For oft his hide had need of patching.

His trowsers, too, were often seatless,
His collar torn and coat 'most sleeveless,
But one thing sure kept away (not far away) all fear,
A new suit came just once a year.

And assessments, too, and annual dueses
Made Keely rich as forty Jewses,
If only rich he could have stayed,
Then all our fears had been allayed.

Now, this Warner's head is a mighty long one,
And the course he takes is not always a wrong one;
I think about this was the view that was taken,
The dentist that's legal is the best to fight Bacon.

The cheaper the Board by law can take them,
The more will be made and the quicker they'd make them.
To fill up the till and keep the fight going,
And pay our attorney was the thing to be doing.

But while one end of the ship was filling
An ugly leak at the other was spilling,
Old members took cold, heavy dues prevailing,
From year to year and never once failing.

To stop that leak there arose a dues doctor,
Who, for love (and for money) could win a backslider,
Forgiving old debts like an old bankrupter,
And starting anew was the thing he was up to.

A good man was he and a mighty wise one,
A father to the young, but to the old he lies some;
Don't speak an untruth, not at all do I mean it,
But lies like a pup when the mother would wean it.

'Tis thus we're brought back to our good old mother,
Brought back, indeed, by an elder brother,
Forgiven and anon with joy reinstated
Among the young ones who were born more (belated).

Thus far I have sought very hard to be sober,
Reviewing old times and living them over;
So don't go and say, I beseech every one of you
That Siddall's play'd smart and tried to make fun of you.

Did ever you meet with an old, old acquaintance
Whose face you'd not seen and whose name you'd not mention'd
For long, long years without first recollecting
Some silly performance you'd not been expecting?

So pardon, I pray you, my seeming great folly,
I'm ashamed of myself, I fear I've been jolly
Over much that was sad and sorely perplexing,
Or outrageously mean and thoroughly vexing.

That things we have found hard enough in the day of it
Are nothing when past is about the way of it;
So then, cheer up my desponding brother,
Though sad to-day you may laugh to-morrow.
If, but an honest day you have made of it,
Then, why in the world should you be afraid of it?

